# Supplementary material for: Portable Rabies Virus Sequencing in Canine Rabies Endemic Countries Using the Oxford Nanopore MinION
Source: Viruses. 2020 Nov 4;12(11):1255. doi: 10.3390/v12111255 (PMC7694271; doi:10.3390/v12111255)
Supplement: Supplementary file 1 [file viruses-12-01255-s001.zip › Supplement Legends.docx]

**Supplementary Material**

**Figure S1 Examples of indels in homopolymer regions of consensus sequences generated from MinION data.** Two insertions (top) and one deletion (bottom) in homopolymers can be seen in the alignment figures. Each row represents a consensus sequence produced by Nanopore sequencing or reference sequence; reference sequence is underlined by the blue bar. Image was taken from alignment visualization in Geneious software.

**Figure S2 Sequence accuracy after different methods of consensus generation for MinION sequencing data.** Number of differences in the nucleoprotein gene (1353 nt) for raw consensus (Raw Consensus), consensus after polishing with raw reads using Nanopolish (Polished Consensus), and polished consensus after manual correction of indels in homopolymer regions (Manual Correction) compared to consensus generated by Sanger sequencing. SNPs, indels, and/or ambiguous bases were all included in the calculation of differences. Each dot represents one sequence. Sequences with over 5,000× read depth are not shown.

**Table S1. Details of samples sequenced in this study.** NT not tested. NA not applicable. Minimum coverage levels are shown. * indicates partial sequence was generated for that sample.

**Table S2. Cost estimate of MinION sequencing of rabies virus amplicons.** Prices based on reagents purchased in the United States as advertised on the Oxford Nanopore website at the time of sequencing (2018). Bulk price of $500 per flow cell was used. Shipping prices are not included. Prices may vary substantially depending on location and product availability.
