# Supplementary figures and images for: Portable Rabies Virus Sequencing in Canine Rabies Endemic Countries Using the Oxford Nanopore MinION
Source: Viruses. 2020 Nov 4;12(11):1255. doi: 10.3390/v12111255 (PMC7694271; doi:10.3390/v12111255)

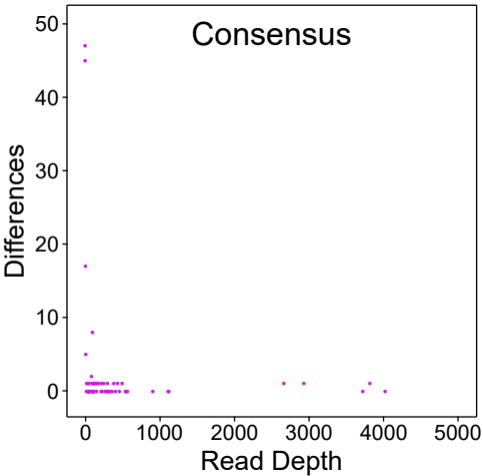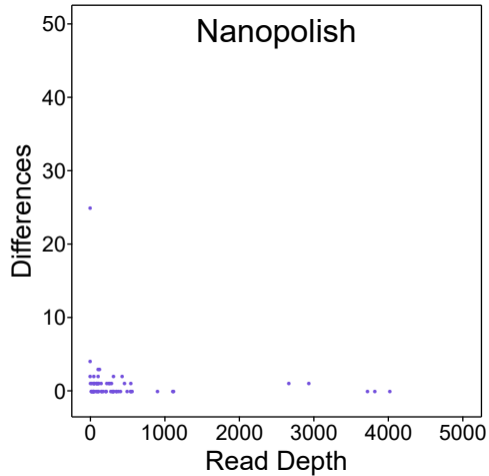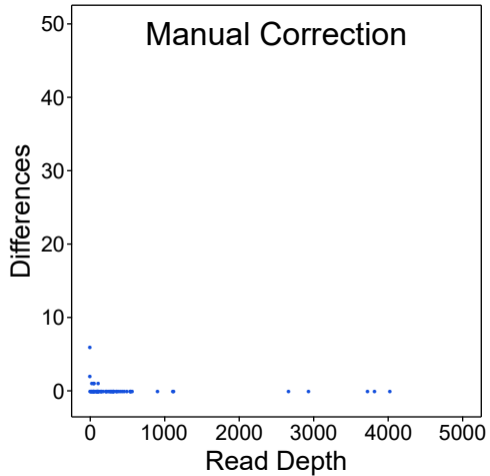

Supplement: Supplementary file 1 [file viruses-12-01255-s001.zip › FigS2.pdf]
