# Supplementary material for: Portable Rabies Virus Sequencing in Canine Rabies Endemic Countries Using the Oxford Nanopore MinION
Source: Viruses. 2020 Nov 4;12(11):1255. doi: 10.3390/v12111255 (PMC7694271; doi:10.3390/v12111255)
Supplement: Supplementary file 1 [file FigS1.pdf › FigS1.pdf]

1,350 1,360 1,370

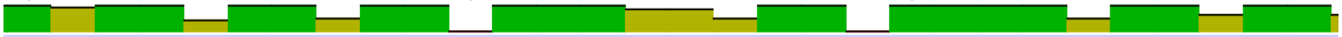

A	C	C	T	G	G	G	T	C	T	-	C	C	C	A	A	A	C	T	-	G	G	G	G	G	A	A	A	T	A
A	C	C	T	G	G	G	T	C	T	-	C	C	C	A	A	A	C	T	-	G	G	G	G	A	A	A	A	T	A
A	C	C	T	G	G	G	T	C	T	-	C	C	C	A	A	A	C	T	-	G	G	G	G	G	A	A	A	T	A
A	C	C	T	G	G	G	T	C	T	-	C	C	C	A	A	A	C	T	-	G	G	G	G	A	A	A	A	T	A
A	C	C	T	G	G	G	T	C	T	-	C	C	C	A	A	A	C	T	-	G	G	G	G	G	A	A	A	T	A
A	C	C	T	G	G	G	T	C	T	-	C	C	C	A	A	A	C	T	-	G	G	G	G	A	A	A	A	T	A
A	C	C	T	A	G	G	C	C	T	-	C	C	C	A	A	A	C	T	G	G	G	G	G	G	A	A	G	T	A
A	C	C	T	A	G	G	C	C	T	-	C	C	C	A	A	A	C	T	-	G	G	G	G	G	A	A	G	T	A
A	C	C	T	G	G	G	T	C	T	-	C	C	C	A	A	A	C	T	-	G	G	G	G	G	A	A	G	T	A
A	C	C	T	G	G	G	T	C	T	-	C	C	C	A	A	A	C	T	-	G	G	G	G	G	A	A	G	T	A
A	C	C	T	G	G	G	T	C	T	-	C	C	C	G	A	A	C	T	-	G	G	G	G	G	A	A	G	T	A
A	C	C	T	G	G	G	T	C	T	C	C	C	C	G	A	A	C	T	-	G	G	G	G	G	A	A	G	T	A
A	U	C	U	C	G	G	U	C	U	-	C	C	C	A	A	G	C	U	-	G	G	G	G	G	A	A	G	U	A

A	C	C	T	A	G	G	C	C	T	-	C	C	C	A	A	G	C	T	-	G	G	G	G	A	A	A	G	T	A
A	C	C	T	A	G	G	C	C	T	-	C	C	C	A	A	G	C	T	-	G	G	G	G	A	A	A	G	T	A
A	C	C	T	A	G	G	C	C	T	-	C	C	C	A	A	G	C	T	-	G	G	G	G	A	A	A	G	T	A
A	C	C	T	A	G	G	C	C	T	-	C	C	C	A	A	G	C	T	-	G	G	G	G	A	A	A	G	T	A
A	C	C	T	A	G	G	C	C	T	-	C	C	C	A	A	G	C	T	-	G	G	G	G	A	A	A	G	T	A
A	C	C	T	A	G	G	C	C	T	-	C	C	C	A	A	G	C	T	-	G	G	G	G	A	A	A	G	T	A
A	C	C	T	A	G	G	C	C	T	-	C	C	C	A	A	G	C	T	-	G	G	G	G	A	A	A	G	T	A
A	C	C	T	A	G	G	C	C	T	-	C	C	C	A	A	G	C	T	-	G	G	G	G	A	A	A	G	T	A
A	C	C	T	A	G	G	C	C	T	-	C	C	C	A	A	G	C	T	-	G	G	G	G	A	A	A	G	T	A
A	C	C	T	A	G	G	C	C	T	-	C	C	C	A	A	G	C	T	-	G	G	G	G	A	A	A	G	T	A
A	C	C	T	C	G	G	T	C	T	-	C	C	C	A	G	A	C	T	-	G	G	G	G	G	A	A	G	T	A
A	C	C	T	C	G	G	T	C	T	-	C	C	C	A	G	A	C	T	-	G	G	G	G	G	A	A	G	T	A

880 890 900

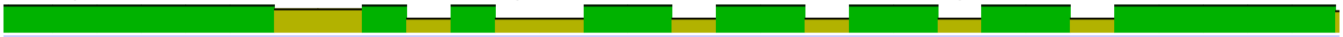

G	G	A	G	T	T	G	G	T	C	A	A	G	A	A	G	A	G	A	G	A	A	G	A	G	T	G	T	C	T
G	G	A	G	T	T	G	G	T	C	A	A	G	A	A	G	A	G	A	G	A	A	G	A	G	T	G	T	C	T
G	G	A	G	T	T	G	G	T	C	A	A	G	A	A	G	A	G	A	G	A	A	G	A	G	T	G	T	C	T
G	G	A	G	T	T	G	G	T	C	A	A	G	A	A	G	A	G	A	G	A	A	G	A	G	T	G	T	C	T
G	G	A	G	T	T	G	G	T	C	A	A	G	A	A	G	A	G	A	G	A	A	G	A	G	T	G	T	C	T
G	G	A	G	T	T	G	G	T	C	A	A	G	A	A	G	A	G	A	G	A	A	G	A	G	T	G	T	C	T
G	G	A	G	T	T	A	G	T	C	A	A	G	A	A	A	A	G	A	G	A	G	G	A	G	T	G	T	C	T
G	G	A	G	T	T	A	G	T	C	A	A	G	A	A	A	A	G	A	G	A	G	G	A	G	T	G	T	C	T
G	G	A	G	T	T	G	G	T	C	A	A	G	A	A	A	A	G	A	G	A	A	G	A	G	T	G	T	C	T
G	G	A	G	T	T	G	G	T	C	A	A	G	A	A	A	A	G	A	G	A	A	G	A	G	T	G	T	C	T
G	G	A	G	T	T	G	G	T	C	A	A	G	A	A	A	A	G	A	G	A	A	G	A	G	T	G	T	C	T
G	G	A	G	T	T	G	G	T	C	A	A	G	A	A	A	A	G	A	G	A	A	G	A	G	T	G	T	C	T
G	G	A	G	U	U	G	G	U	C	A	A	G	A	A	A	A	G	A	G	A	A	G	A	G	U	G	U	C	U

G	G	A	G	T	T	G	G	T	T	A	G	A	A	A	G	A	G	G	G	A	G	G	A	A	T	G	T	C	T
G	G	A	G	T	T	G	G	T	T	A	G	A	A	A	G	A	G	G	G	A	G	G	A	A	T	G	T	C	T
G	G	A	G	T	T	G	G	T	T	A	G	A	A	A	G	A	G	G	G	A	G	G	A	A	T	G	T	C	T
G	G	A	G	T	T	G	G	T	T	A	G	A	A	A	G	A	G	G	G	A	G	G	A	A	T	G	T	C	T
G	G	A	G	T	T	G	G	T	T	A	G	A	A	A	G	A	G	G	G	A	G	G	A	A	T	G	T	C	T
G	G	A	G	T	T	G	G	T	T	A	G	A	A	A	G	A	G	G	G	A	G	G	A	A	T	G	T	C	T
G	G	A	G	T	T	G	G	T	T	A	G	A	A	A	G	A	G	G	G	A	G	G	A	A	T	G	T	C	T
G	G	A	G	T	T	G	G	T	T	A	G	A	A	A	G	A	G	G	G	A	G	G	A	A	T	G	T	C	T
G	G	A	G	T	T	G	G	T	T	A	G	A	A	A	G	A	G	G	G	A	G	G	A	A	T	G	T	C	T
G	G	A	G	T	T	G	G	T	T	A	G	A	A	A	G	A	G	G	G	A	G	G	A	A	T	G	T	C	T
G	G	A	G	T	T	G	A	T	C	A	G	G	A	A	-	A	G	G	G	A	A	G	A	A	T	G	T	C	T
G	G	A	G	T	T	G	A	T	C	A	G	G	A	A	A	A	G	G	G	A	A	G	A	A	T	G	T	C	T
